# Dual-Functional Nano-Functionalized Titanium Scaffolds to Inhibit Bacterial Growth and Enhance Osteointegration

**DOI:** 10.3390/nano11102634

**Published:** 2021-10-07

**Authors:** Giovanna Calabrese, Domenico Franco, Salvatore Petralia, Francesca Monforte, Guglielmo Guido Condorelli, Stefano Squarzoni, Francesco Traina, Sabrina Conoci

**Affiliations:** 1 Department of Chemical, Biological, Pharmaceutical and Environmental Sciences, University of Messina, 98166 Messina, Italy; dfranco@unime.it; 2 Department of Drug Science and Health, University of Catania, 95125 Catania, Italy; salvatore.petralia@unict.it; 3 Department of Chemical Science, University of Catania, 95125 Catania, Italy; marzia.monforte@gmail.com (F.M.); guido.condorelli@unict.it (G.G.C.); 4 CNR—Institute of Molecular Genetics “Luigi Luca Cavalli-Sforza”, 40136 Bologna, Italy; squarzoni@area.bo.cnr.it; 5 IRCCS Istituto Ortopedico Rizzoli, 40136 Bologna, Italy; traina.francesco@gmail.com; 6 Department of DIBINEM, University of Bologna, 40136 Bologna, Italy; 7 Istituto per la Microelettronica e Microsistemi, Consiglio Nazionale delle Ricerche (CNR-IMM), Ottava Strada n.5, I-95121 Catania, Italy

**Keywords:** γFe_2_O_3_, TiO_2_, nano-functionalization, titanium alloy, osteointegration, osteoinduction, antibacterial properties, biocompatibility

## Abstract

Implantable biomaterials play a key role for the success of orthopedic surgery procedures. However, infections remain one of the most damaging post-operative complications that lead to the implant failure. Recently, several approaches have been proposed to avoid or manage implant-associated infections. Among these, an appropriate surface functionalization to confer intrinsic antibacterial properties preserving the osteo-integration ability represents an appealing strategy for the development of innovative implant materials. Titanium and its alloys are the most used materials for manufacturing of both articular and bone skull prostheses as well as dental implants. However, to date there is still a significant clinical need to improve their bioactivity, osseointegration and antibacterial activity. In this study, titanium biomimetic scaffolds are prepared by nano-functionalization with TiO_2_ (Ti_TiO_2_) and γFe_2_O_3_ (Ti_γFe_2_O_3_). Both cytocompatibility and antibacterial activity have been evaluated. Data show that both nano-functionalized scaffolds exhibit a good antibacterial activity towards *Staphylococcus aureus*, reducing colony number to 99.4% (Ti_TiO_2_) and 99.9% (Ti_γFe_2_O_3_), respectively. In addition, an increase of both human adipose-derived mesenchymal stem cells (hADSCs) cell proliferation (up to 4.3-fold for Ti_TiO_2_ and 3.7-fold for Ti_γFe_2_O_3_) and differentiation has been observed. These data suggest that these nano-functionalized titanium substrates represent promising prototypes for new antimicrobial and osteoconductive biomaterials to be used in the orthopedic field to reconstruct significant bone defect.

## 1. Introduction

In recent years, the number of people undergoing surgery for osteoarticular problems, such as osteoarthritis and osteoporotic fractures, is greatly grown due to an overall increase in life expectancy. Biomaterials play a key role for the success of orthopedic surgery procedures. However, despite significant advances in the patient’s quality of life, implant-associated infections remain one of the most damaging post-operative complications [1]. Actually, the prosthesis insertion in the body is associated to the risk of microbial infection and osteomyelitis occurrence causing implant failure [2,3] inducing surgery procedures to replace the infected prosthesis and, in the worst cases, limb amputation. Some evidences showed that the infection rate in patients with open fractures is about 20%, but it can be higher than 50% in much more severe cases [4]. Bacterial contamination during the surgery includes several sources such as operating environment, surgical equipment, clothing worn by medical and paramedical personnel and resident bacteria on the skin and mucous membranes of the patient himself [5]. Most contaminations belong to the Gram-positive Staphylococci family such as *Staphylococcus aureus* (*S. aureus*) and *Staphylococcus epidermidis*, and Gram-negative bacilli including *Pseudomonas aeruginosa* and *Escherichia coli* [6]. The infections are caused by bacteria adhesion on the implant surface and subsequent biofilms formation [7]. This leads to many complications including chronic infections, resistance to treatment with antibiotics and chronic inflammatory response at the site of the biofilm itself [7,8]. Therefore, the inhibition of both bacterial surface adhesion and proliferation are the main points to be considered to design antibacterial materials. The elements influencing bacterial adhesion to the implant surface are the physical properties such us the material microstructure, hydrophobicity, superficial charge and surface roughness [9].

Titanium (Ti) and its alloys are the most used materials for orthopedic implants due to their exceptional mechanical and chemical properties, good biocompatibility, corrosion resistance, good bone affinity and osteoconductivity [10,11]. Several approaches are reported in the literature to enhance the bone tissue regeneration of Ti-based devices by the development of appropriate trabecular topography, bio-coating [12] or specific pore structure [13]. The internal pore structure also affects the mechanical stiffness of the material [14,15,16] thereby being instrumental for the design of novel long-lasting implants.

However, although Ti shows these excellent properties, to date there is still a significant clinical need to improve its antibacterial properties preserving osteo-integration ability. Therefore, the development of new biomaterials with bactericidal/osteoinductive function is still a challenge. Several approaches have been proposed in the literature for Ti substrates mainly involving: (1) the use of materials with intrinsic antibacterial properties (Ag, Cu, Zn and polymers) [17,18,19] and (2) surface functionalization by specific coating [20,21] or surface structuring [22,23]. In this context, nanotechnology is a powerful tool allowing the design of innovative biomaterials by playing on chemistry of materials and size. Using nanostructured materials, free-living or surface-associated bacteria (biofilm) can be inhibited by an ad-hoc modulation of the biomaterial chemical-physical properties (size, surface morphology, charge and zeta potential) [24]. Antimicrobial activity of nano-materials mainly involves both oxidative stress induction and metal ion release. The first derived from free radical’s generation leading to damage on cellular wall and nucleic acids. The release of metal ions (positively charged) to the surface of bacteria (negatively charged) enhances antimicrobial activity by destabilization of cell membrane and, inside cells, by proteins denaturation (especially ribosome proteins) [25]. However, often antibacterial effects are associated to cytotoxicity with a consequent increase of health risk [26,27,28]. 

In particular, metal or metal oxide nanoparticles (NPs) are increasing their use as alternative to antibiotics due to their broad spectrum of action against both gram +(ve) and gram −(ve) bacteria [15]. Among these, TiO_2_ NPs are among the most studied due to their efficiency and versatility based on photo-catalytic mechanism upon light irradiation depending on crystal structure, doping metals and irradiation wavelength [29,30]. The bactericidal mechanism of TiO_2_ is mainly due to Reactive Oxygen Species (ROS) generation and lipid oxidation of bacterial cell wall. The ROS generation has been also found in the absence of light in presence of species undergoing catalytic decomposition (like H_2_O_2_) [31,32]. Among the form of nanostructures, titania nanotubes have been extensively studied for osteogenesis highlighting that a combination of nano- and microscale roughness at the implant surface of these nano-systems can promote relevant bioactivity [33].

Iron (III) oxide NPs are also appealing to nano-materials for tissue regeneration, since iron is an essential micro nutrient for cell growth and can also exhibit magnetic properties promoting cell proliferation [34,35]. Additionally, they can show antibacterial effects both as Fe_2_O_3_ and as Fe_3_O_4_ due to multiple mechanisms including ROS species, superoxide radical’s generation and singlet oxygen formation [36,37]. However, despite the advantages described above, chemically synthesized iron oxide nanoparticles agglomerate and possess reduced stability [38], requiring post-synthesis modification [39]. Therefore, the integration of nano-materials with new properties into Ti biomimetic scaffolds can be an effective strategy to develop innovative biomaterials for tissue engineering. 

In this paper we proposed the preparation of new dual functional material with antibacterial and osteo-integration ability based on surface nano-functionalization of Ti alloy with TiO_2_ and γFe_2_O_3_ NPs (Ti_TiO_2_ and Ti_γFe_2_O_3_) by wet chemistry. Both the antibacterial properties towards *S. aureus* and the cell growth and differentiation using hADSCs have been assessed by in vitro studies and discussed.

## 2. Materials and Methods

### 2.1. Chemicals and Materials

All chemicals were obtained from commercial sources at the highest possible purity and were used as received. All solvents used were spectrophotometric grade. Milli-Q-grade water was used in all preparations. Ti supports were provided by Istituto Ortopedico Rizzoli (Bologna, Italy). The supports are made of trabecular Ti_6_Al_4_V alloy and circular shaped with diameter of 8 mm and height of 4 mm.

### 2.2. Titanium Scaffolds Nano-Functionalization Procedure

(a) Nano-functionalization with TiO_2_ (Ti_TiO_2_). Ti supports were dipped in a titanium isopropoxide (0.1 M) isopropyl alcohol solution for 5 min and heated in air at 600 °C for three times. After that, the Ti_TiO_2_ scaffolds were rinsed with isopropyl alcohol and dried under nitrogen.
(b) Nano-functionalization with γFe_2_O_3_ NPs (Ti_γFe_2_O_3_). Ti supports were cleaned in ethanolic ultrasound bath for 10 min and after, they were placed in a quartz tube containing ethyl alcohol, iron (III) acetylacetonate and acetone as photosensitizers. After 15 min of degassing with N_2_, the solution was irradiated with four lamps (254 nm 16 W) for 90 min. NaOH 1 M was added to the resulting yellow solution and stirred in air for 60 min. Finally, the red-brown Ti_γFe_2_O_3_ substrates were rinsed three times with deionized water and dried with nitrogen flow.

### 2.3. Physical-Chemical Characterization of Titanium Scaffolds

The morphological and microstructural properties of nano-derivatised Ti scaffold surfaces were evaluated by Scanning Electron Microscopy (SEM-EDX) performed on a SEM-LEO 438 VP (Carl Zeiss AG, Oberkochen, Germany) and a Zeiss EVO MA10 (Carl Zeiss AG, Oberkochen, Germany) equipped with a LaB6 electron gun. Transmission electron microscopy (TEM) analysis was performed using the bright field in conventional parallel beam (CTEM) mode (BF). An ATEMJEOL JEM-2010 (Pleasanton, CA, USA) equipped with a 30-mm^2^ window energy-dispersive X-rays spectrometer was used. Atomic force measurements (AFM) were performed using a Solver P47 (NT-MDT instrument, Moscow, Russia) in contact mode. We used standard silicon AFM probes (NT-MDT instrument, Russia) having cantilever force constant in the 0.01–0.5 Nm range. The zeta potential was evaluated using a ZetaSizer NanoZS90 (Malvern Panalytical Ltd., Cambridge, UK), equipped with a 633 nm laser, at the scattering angle of 90 °C and 25 °C temperature. Z-potential measurements were performed in aqueous dispersion at pH 7.2. Each measurement was performed three times.

### 2.4. In-Vitro Tests

(a) Cell culture and proliferation analysis.

hADSCs were acquired from Lonza (Lonza Group Ltd., Basel, Switzerland) and cultured as previously reported [40] until 80–90% of confluence.

Trypan blue test. 1 × 10^6^ cell were plated on Ti scaffolds (Ti_CTRL (not-treated Titanium substrate), Ti_TiO_2_, Ti_γFe_2_O_3_) in culture medium and incubated in a humidified atmosphere containing 5% CO_2_ at 37 °C for 1, 3 and 7 days. After, hADSCs were detached from Ti-derivatized supports with trypsin (Sigma-Aldrich, Milan, Italy), stained with trypan blue (Termo Fisher Scientific, NYSE: TMO) and counted by using a Leica DMI 4000B fluorescence microscope (Leica Microsystems Srl, Milano, Italy).

4′,6-Diamidino-2-Phenylindole (DAPI, Thermo Fischer Scientific, NYSE: TMO, Waltham, MA, USA) staining. 1 × 10^6^ hADSCs were cultured on each scaffold for 1, 3 and 7 days, fixed in 4% PFA (paraformaldehyde) and washed three times in PBS (Sigma-Aldrich, Milan, Italy). After, each scaffold was permeabilized in 0.3% Triton X-100 for 10 min, washed in PBS and the nuclei stained with DAPI (1:5000) in PBS for 5 min. Finally, the images (20 for each scaffold) were acquired using a Leica DMI 4000B fluorescence microscope and the nuclei counted by Fiji Image J recognition software. The proliferation rates differences were assessed using One-way ANOVA test with Holm test as post-hoc for multiple comparisons. Each biological test was conducted in triplicate for each time points.

(b) Osteogenic Differentiation.

Alizarin Red S (ARS) staining quantification assay. ARS staining (Sciencell, Italy) was performed to evaluate calcium deposits in cells culture according to the manufacturer’s instructions. Specifically, 1 × 10^6^ hADSCs were cultured on each scaffold for 1, 3 and 7 days in 6-well plates. After that, the cells on the scaffolds were washed with PBS and fixed in 4% PFA at room temperature for 15 min and then washed twice with excess dH_2_O prior to addition of 1 mL of 40 mM ARS (pH 4.1) per well. The scaffolds were incubated at room temperature for 20 min under shaking. After aspiration of the unincorporated dye, they were washed four times with 4 mL dH_2_O while shaking for 5 min, re-aspirated and stored at −20 °C prior to dye extraction. For the quantification of ARS, 800 µL 10% (*v/v*) acetic acid was added to each well, and the plate was incubated at room temperature for 30 min with shaking. The cells were then detached from the scaffolds, transferred with 10% (*v/v*) acetic acid to a 1.5-mL microcentrifuge tube and vortexed for 30 s. After, the samples were sealed with parafilm, heated to 85 °C for 10 min and transferred to ice for 5 min. They were then centrifuged at 20,000× *g* for 15 min and 500 µL of the supernatant was removed to a new 1.5-mL microcentrifuge tube. Then 200 µL of 10% (*v/v*) ammonium hydroxide was added to neutralize the acid. In total, 150 µL of the supernatant for each sample were read in triplicate at 405 nm by a Microplate Reader (Biotek, Waltham, MA, USA). 

Alkaline phosphatase (ALP) activity evaluation. ALP activity was evaluated by colorimetric assay performed on cell culture supernatants. Specifically, 1 × 10^6^ hADSCs were cultured on each scaffold for 1, 3 and 7 days and the culture media collected and used to measure ALP activity. This was enzymatically determined using an alkaline phosphatase assay kit (ab83369, Abcam, Cambridge, UK) according to the manufacturer’s instructions. The ALP level was measured by reading the absorbance at 405 nm by a Microplate Reader (Biotek, USA). All experiments were performed in triplicate.

(c) Bacteria strain and viability assay.

*S. aureus* (ATCC 29213) was purchased from American Type Culture Collection (LGC Promochem, Milan, Italy) and cultured in Tryptone Soya Broth (TSB, Sigma-Aldrich, Milan, Italy). Ti scaffolds were sterilized in autoclave at 121 ± 1 °C for 20 min and placed in 24-well plates (one scaffold per well). Bactericidal activity was evaluated according to Hu et al. [41] with some modifications. Briefly, a colony of *S. aureus* cultured overnight in Tryptone Soya Agar (TSA, Sigma-Aldrich, Milan, Italy), was inoculated in Mueller–Hinton Broth (MHB, Sigma-Aldrich, Milan, Italy) and incubated at 150 rpm on a rotary shaker for 6–8 h at 37 °C. After incubation period, the bacteria suspension (10^7^ cells/mL) was placed onto the titanium scaffold surfaces with a 60 µL/cm^2^ density, and incubated in a humidified atmosphere at 37 °C for 24 h. Then, the scaffolds were placed in tubes containing phosphate-buffered saline (PBS) solution and, vigorously stirred for 60 s to detach the bacteria from the coating surface. Finally, a Colony Forming Units (CFU) assay was performed by using an untreated titanium scaffold as control, as following. Specifically, 100 µL of bacterial suspension were serially diluted in 900 µL of PBS, 100 µL of each dilution spread on solid medium and incubated overnight at 37 °C. After incubation, colonies in the range of 30–300 were counted to determine the number of CFU:

$$\mathrm{CFU}=\frac{\left(\mathrm{number}\;\mathrm{of}\;\mathrm{colonies}\right)}{\mathrm{volume}\;\left(0.1\;\mathrm{mL}\right)\times \mathrm{dilution}\;\mathrm{factor}}$$

The bacterial adhesion on the surface TiO_2_ e γFe_2_O_3_ nano-functionalized scaffolds were evaluated by live/dead cell staining kit (BacLight™ Bacterial Viability Kit, ThermoFischer Scientific, NYSE: TMO). Briefly, after bacteria incubation, the scaffolds were stained with a mixture of SYTO 9 (green-fluorescent staining viable cells) and propidium iodide (red-fluorescent staining dead cells) for 15 min. After, they were visualized under fluorescence microscopy by using Leica DMRE epifluorescence microscope (Leica Microsystems, Heerbrugg, Switzerland) with Leica C Plan 63xobjective and BP 515–560 nm excitation filter in combination with a LP 590 nm suppression filter.

(d) ROS evaluation.

For ROS evaluation, bacteria were detached, washed twice with PBS, stained with 2′, 7’-dichlorodihydrofluorescein diacetate (H_2_DCFDA, Sigma-Aldrich, Milan, Italy) at 10 µM final concentration and incubated at 37 °C for 20 min. After the incubation time, bacteria were washed twice with PBS, to remove the excess of dye, and finally images were acquired by using a Leica DMRE epifluorescence microscope with Leica C Plan 100x objective and BP 515–560 nm excitation filter in combination with a LP 590 nm suppression filter.

(e) Statistical analysis.

Data were analyzed either as raw data or as mean ± standard error (SE), as appropriate. Differences between the two time points and the different types of scaffolds were evaluated by using one- and two-way ANOVA with post-hoc Holm test, where appropriate. The values *p* < 0.05 were considered to be significant.

## 3. Results and Discussion

### 3.1. Morphological and Microstructural Analyses of Titanium Scaffolds

**Figure 1:**
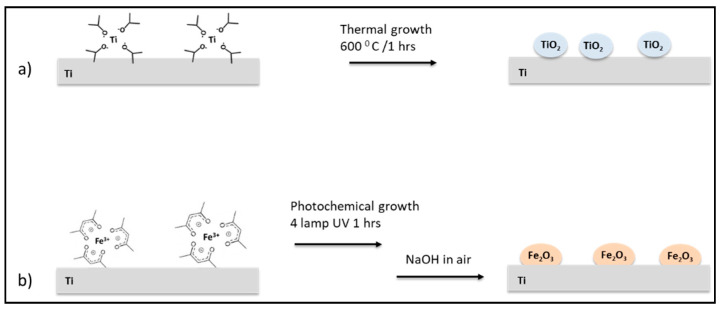
Schematic representation of titanium scaffold surface nano-functionalization processes with (**a**) TiO_2_ and (**b**) γFe_2_O_3_.

Both Ti_TiO_2_ and Ti_γFe_2_O_3_ scaffolds were prepared by surface in-situ growth with wet chemistry. Ti-isopropoxide precursor were converted in nanosized TiO_2_ on scaffold surface trough thermal growth (Figure 1a). Similarly, Ti_γFe_2_O_3_ scaffold were obtained with a two-steps approach based on the photochemical reaction of Fe(acac)_3_ precursor in the presence of photosensitizers, followed by pH-basic oxidation to obtain nanosized γFe_2_O_3_ on the scaffold surface (Figure 1b).

**Figure 2:**
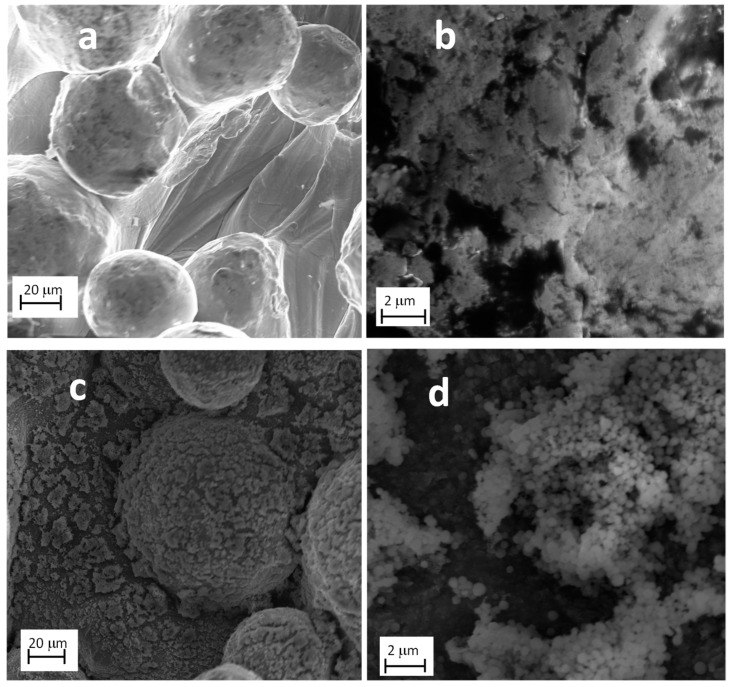
Representative SEM images for Ti scaffolds (**a**,**b**) not treated samples and (**c**,**d**) Ti_TiO_2_ scaffolds.

Both proposed synthetic methods were based on chemical reactions converting specific precursors (Ti(isopropoxide)_4_ and FeIII(acac)_3_) into TiO_2_ and γFe_2_O_3_ NPs at the scaffold surface. The nano-derivatized biomimetic scaffolds were morphologically and chemically characterized trough AFM, SEM, EDX and TEM inspections. Figure 2 reports SEM images of the biomimetic scaffolds before (Figure 2a,b) and after TiO_2_ nano-functionalization (Figure 2c,d). The images clearly demonstrate the presence of TiO_2_ nanostructures with dimensions between 300 nm and 800 nm in diameter (Figure 2c,d). 

**Figure 3:**
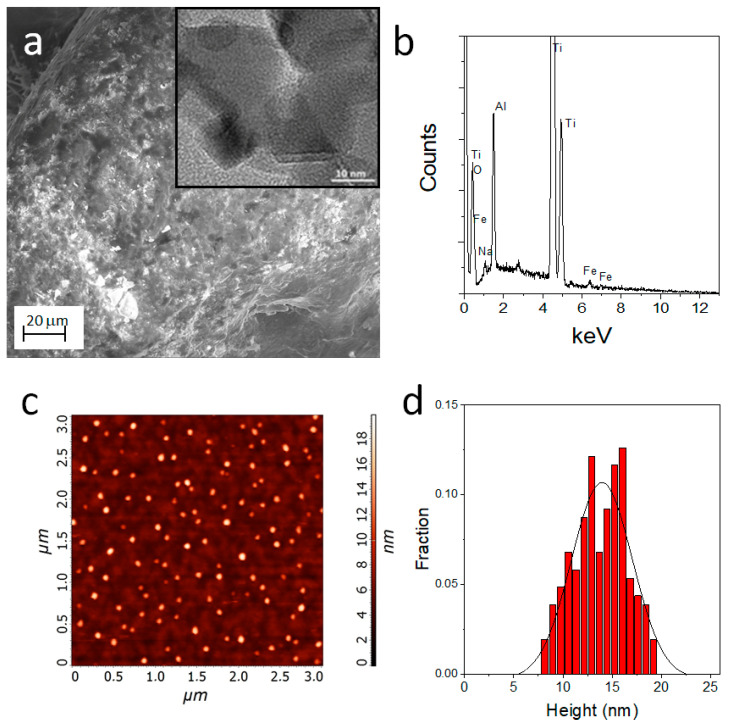
(**a**) Representative SEM image of Ti_γFe_2_O_3_ scaffolds; Insert TEM image for Ti_γFe_2_O_3_; (**b**) EDX data of Ti_γFe_2_O_3_; (**c**) AFM images of γFe_2_O_3_ NPs on flat Si(100); (**d**) statistical analysis performed on an area of 5 μm × 5 μm of the observed feature heights.

The EDX analysis indicates a significant increase of the O peak (0.55 KeV) compared to the untreated Ti_CTRL titanium scaffold (see Figure S1), confirming the presence of TiO_2_ nanostructures. In the case of Ti_γFe_2_O_3_ it was not possible to detect the presence of γFe_2_O_3_ NPs in the SEM images because of their nanosize (Figure 3a). The presence of iron was assessed through EDX analysis which showed the specific peaks for the iron species at 6.4 and 7.1 KeV (Figure 3b). The size of the γFe_2_O_3_ NPs was obtained combining TEM analysis and AFM measurements. TEM (insert of the Figure 3b) performed on the powders formed during the scaffold functionalization showed the presence of NPs with a diameter in the 10–20 nm range. The diffraction analysis indicates the diagnostic d-spacing values for the maghemite (γFe_2_O_3_): 2.95 Å (28%), 2.54 Å (100%), 2.09 Å (20%), 1.73 Å (10%), 1.64 Å (25%) and 1.47 Å (42%) (see Figure S2). AFM (Figure 3c), performed on samples obtained on flat Si(100) substrates, indicate the presence of γFe_2_O_3_ NPs with an average size of about 14 ± 3 nm (Figure 3d). The average value of the size obtained from the AFM is higher than the typical TEM sizes likely due to the slight NPs aggregation occurring during NPs deposition. These values are in accordance with the data reported in literature and definitively confirm the presence of γFe_2_O_3_ NPs.

In addition, we performed Z-potential investigation on TiO_2_ and γFe_2_O_3_ NPs in solution. Our results indicate the presence of negative charged nanoparticles with Z-potential values of −11.8 ± 1.4 mV for TiO_2_ and −39.2 ± 0.9 mV for γFe_2_O_3_. Surface negative charges that can promote the adsorption of hydrophilic proteins (vitronectin, fibronectin, etc.) leading to increased bone cell adhesion [42].

### 3.2. hADSCs Proliferation and Differentiation Evaluation on Titanium Scaffolds

**Figure 4:**
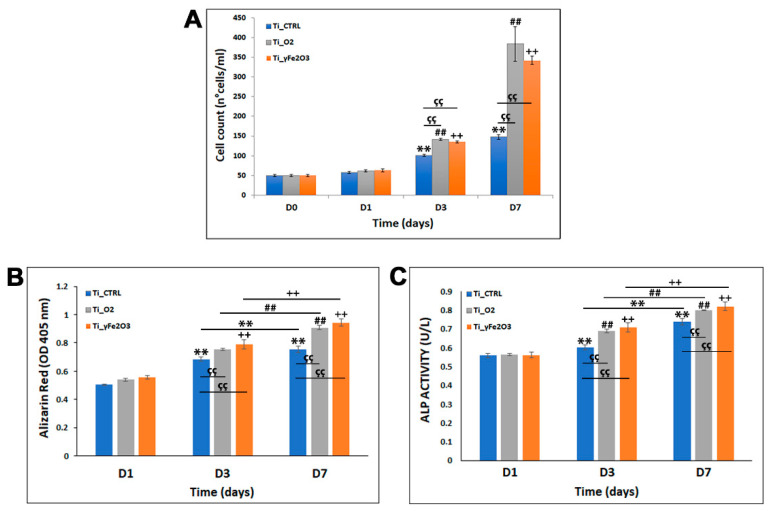
Cell proliferation analysis of hADSCs cultured on Ti_CTRL, Ti_TiO_2_ and Ti_γFe_2_O_3_ scaffolds, for 7 days. (**A**) Trypan blue cell count at day 0 (D0 = plating day), day 1 (D1 = 24 h from plating), day 3 (D3 = 3 days from plating) and day 7 (D7 = 7 days from plating). Data are reported as mean ± standard deviation obtained on 3 scaffolds. (**B**) Alizarin Red S staining after 1, 3 and 7 days of cell culture. Data are shown as mean ± standard deviation. (**C**) ALP activity after 1, 3 and 7 days of cell culture. Data are shown as mean ± standard deviation. **, ^##^, ^çç^, ^++^ *p* < 0.01 show significant differences between the different time points and scaffolds, as reported by the Holm post-hoc test.

To gain insight on the osteo-integration ability of both Ti_TiO_2_ and Ti_γFe_2_O_3_, cell proliferation was investigated by observing the viability of hADSCs for 1, 3 and 7 days on nano-functionalized scaffolds (cultured at the same density in parallel cultures) compared to not treated Ti_CTRL scaffold (Figure 4A). The cells were firstly allowed to interact on the scaffold surfaces for 24 h (D1) and Trypan blue assay was performed to measure the proliferation rate at three time points (1, 3 and 7 days—the cell number cultured on the scaffolds at day 0 was used as reference value). Results showed that over a period of 7 days, the proliferation rate of hADSCs on both Ti_TiO_2_ and Ti_γFe_2_O_3_ scaffolds was considerably higher than of Ti_CTRL (Figure 4A) and that cell number increased over time. More specifically, while at D1, hADSCs proliferation rate of both Ti_TiO_2_ and Ti_γFe_2_O_3_ scaffolds was comparable with that of the Ti_CTRL (control) (Ti_TiO_2_ = 62 ± 2.65, Ti_γFe_2_O_3_ = 63 ± 3.87, Ti_CTRL = 58 ± 2.55), after 3 days (D3) the cell numbers on Ti_TiO_2_ and Ti_γFe_2_O_3_ scaffolds were 1,4-fold and 1,3-fold higher than Ti_CTRL control and at D7 these values increased to 2.3-fold and 2.6-fold, respectively, (D3:Ti_TiO_2_ = 142 ± 2.54, Ti_γFe_2_O_3_ = 135 ± 2.62, Ti_CTRL = 101 ± 2.38; D7:Ti_TiO_2_ = 384 ± 43.79, Ti_γFe_2_O_3_ = 342 ± 10.78, Ti_CTRL = 148 ± 5.18).

These data were also confirmed with DAPI staining which, after 24 h of cell growth (D1), showed no significant difference in the cell number in the nano-functionalized scaffolds compared to Ti_CTRL. After 3 and 7 days of culture in both Ti_TiO_2_ and Ti_γFe_2_O_3_ scaffolds a considerable cell number increasing of for 3.7-fold Ti_TiO_2_ and 4.3-fold for Ti_γFe_2_O_3_, compared to the Ti_CTRL control was found (see Figure S3). Contrary, the cell number difference between the two nano-functionalized scaffolds was not significant. 

In addition to the osteo-integration, we also evaluated another important aspect for implantable-devices, the osteogenic differentiation. To this aim, ARS staining quantification and ALP activities after 1, 3, and 7 days of culture on both Ti_TiO_2_ and Ti_γFe_2_O_3_ were measured. Data obtained from ARS staining quantification show a higher calcium deposits content in both Ti_TiO_2_ and Ti_γFe_2_O_3_ scaffolds than Ti_CTRL control at the same incubation times (Figure 4B). In detail, after 3 days (D3) of culture on scaffolds the ARS value increases of about 7% for Ti_TiO_2_ and 11% for Ti_γFe_2_O_3_ respect to the Ti_CTRL control; while, at D7 these values correspond to about 15% for Ti_TiO_2_ and 19% for Ti_γFe_2_O_3_, respectively. On the contrary, not significant difference was found between Ti_TiO_2_ and Ti_γFe_2_O_3_ scaffolds at all times analyzed (*p* > 0.05).

In accordance with ARS staining quantification data, ALP results show values significantly higher in both Ti_TiO_2_ and Ti_γFe_2_O_3_ scaffolds than Ti_CTRL control at the same incubation times (Figure 4C). Specifically, after 3 days (D3) the ALP activities increase of about 9% for Ti_TiO_2_ and 11% for Ti_γFe_2_O_3_ respect to the Ti_CTRL control; while, at D7 these values correspond to about 6% for Ti_TiO_2_ and 8% for Ti_γFe_2_O_3_, respectively. Additionally, in this case, no significant difference was found between Ti_TiO_2_ and Ti_γFe_2_O_3_ scaffolds at all times analyzed (*p* > 0.05). These data together indicate that both nano-functionalizations are able to induce osteogenic differentiation.

**Figure 5:**
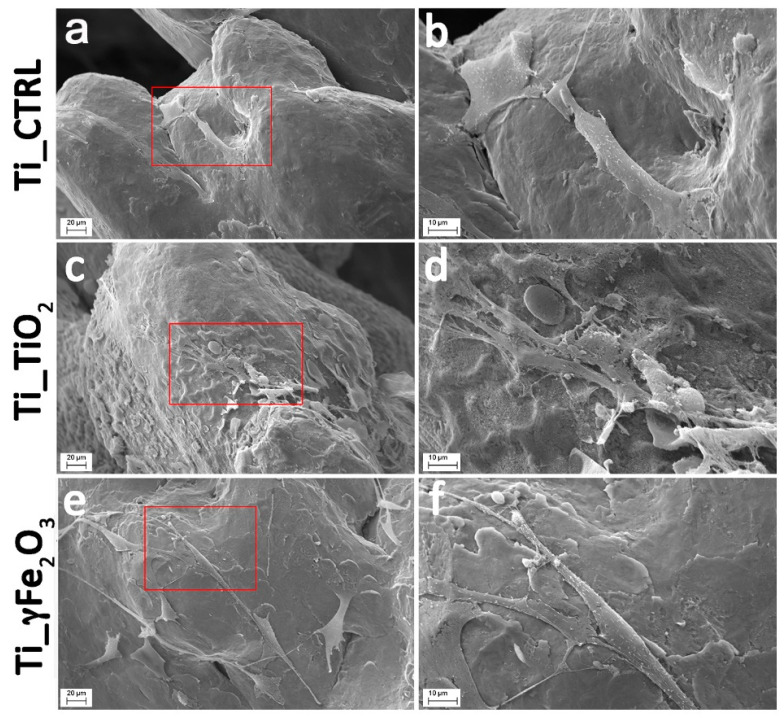
Representative SEM images of Ti_CTRL (**a**,**b**), Ti_TiO_2_ (**c**,**d**) and Ti_γFe_2_O_3_ (**e**,**f**) scaffolds with hADSCs after 7 days from the seeding. Magnification 1000× (**a**,**c**,**e**); magnification 3000× (**b**,**d**,**f**).

After cell culture, the nano-derivatized scaffolds were inspected by SEM to characterize the surface topographical features upon cellular interaction. Figure 5 reports representative SEM images of Ti_CTRL (Figure 5a,b), Ti_TiO_2_ (Figure 4c,d) and Ti_γFe_2_O_3_ (Figure 5e,f) after 7 days of culture. The images visibly show that the cells are able to adhere and proliferate in nano-functionalized titanium scaffolds and that at 7 days after cell seeding, the cells on all scaffolds became even more stretched maintaining the typical morphology of mesenchymal stem cells, characterized by long and thin cellular processes.

The above results highlight that both Ti_TiO_2_ and Ti_γFe_2_O_3_ biomimetic scaffolds are able to promote hADSCs adhesion, proliferation, grow and differentiation, indicating their effective osteo-integrative/inductive ability. This finding is corroborated by several literature evidences showing that the nanostructing of the scaffold combined with surface properties can improve the cell adhesion and proliferation achieving better osseointegration [42,43]. In our case, several factors can be considered to comment the obtained results. Firstly, the biocompatibility of the nano-materials. Actually, both TiO_2_ and γFe_2_O_3_ have been proved to be biocompatible for bone regenerative application [33,34]. Then, the scaffolds surface topography whose biomimetic feature is an important parameter affecting both cell adhesion and proliferation specially during the phase of osseointegration. It has been really observed that even if nanotopography by itself promotes bone cell functions, however the combination of nano- and micro-scale roughness can enhance bioactivity [33]. Our finding shows that both TiO_2_ and γFe_2_O_3_ NPs form nanostructured coatings (300–800 nm for TiO_2_ NPs and 10–20 nm for γFe_2_O_3_ NPs) spread onto the micrometric trabecular microstructure of the pristine Ti substrate. These results, therefore, evidence that a combined micro-nanotopography can promote and induce the cellular integration.

Another important factor for cell–biomaterial interaction is the surface hydrophilicity, that improve adhesion and spread of cells, osteoblastic differentiation and maturation [44]. Both TiO_2_ and γFe_2_O_3_ nano-systems exhibit negative charges at the surface (see above the Z-potential data) indicating the possibility to improve the adsorption of hydrophilic proteins (vitronectin, fibronectin, etc.) leading to increased bone cell adhesion [41].

### 3.3. In Vitro Antibacterial Activity Evaluation

**Figure 6:**
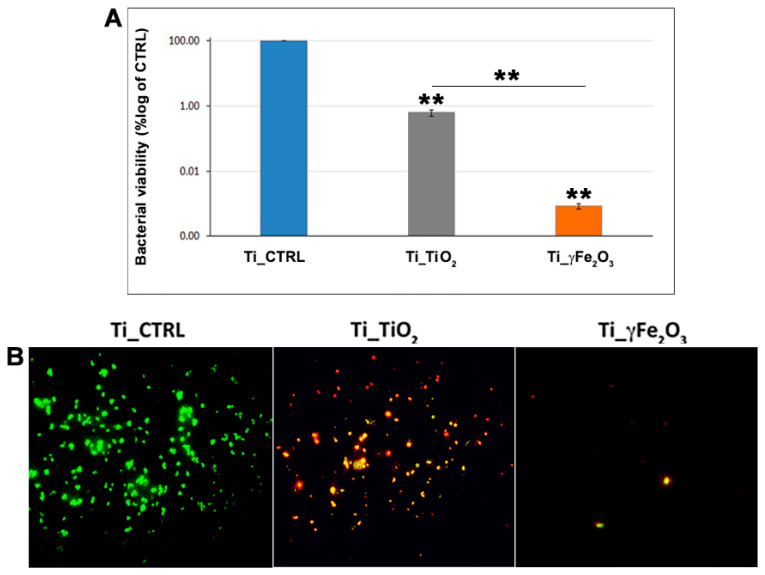
(**A**) CFU assay performed on Ti_TiO_2_ and Ti_γFe_2_O_3_ scaffolds, compared to the Ti_CTRL, after 24 h of *S. aureus* bacteria incubation. The data are expressed as CFU average percentage ± standard deviation of six replicates. ** *p* < 0.01 shows significant differences between the different time points as reported by the Holm post-hoc test. (**B**) Live/Dead staining of the cells adhering to the scaffolds. The green and red stains indicate the presence of live and dead bacteria, respectively.

In order to assess the antibacterial properties of both Ti_TiO_2_ and Ti_γFe_2_O_3_ scaffolds, *S. aureus* bacteria were incubated on them for 24 h, and after CFU assay and live/dead staining were performed. Results are reported in Figure 6. Data highlight a good antibacterial activity for both for Ti_TiO_2_ and Ti_γFe_2_O_3_ scaffolds compared to the Ti_CTRL scaffold. Specifically, the CFU assay reveals that TiO_2_ and γFe_2_O_3_ NPs incorporated on the titanium scaffold surfaces are able to reduce the colony number of about 99.4% for Ti_TiO_2_ (3.2 ± 0.7 × 10^5^ CFU/mL) and 99.9% for Ti_γFe_2_O_3_ (4.3 ± 0.9 × 10^2^ CFU/mL) compared to the Ti_CTRL (3.7 ± 1.4 × 10^7^ CFU/mL) (Figure 6A). In addition, Ti_γFe_2_O_3_ scaffold exhibited a stronger statistically significant antibacterial activity compared to the Ti_TiO_2_ scaffold. These results were in agreement with those of the Live/Dead staining showing the presence of very few living (green fluorescent) and many dead (red fluorescent) cells for Ti_TiO_2_, while almost all of them were red for Ti_γFe_2_O_3_ compared to the Ti_CTRL, demonstrating their strong biocidal activity (Figure 6B). Consequently, it’s possible to deduce that bactericidal effect of γFe_2_O_3_ is both higher and faster compared to TiO_2_. These findings could be related to the different nature of NPs, as well as to the size (TiO_2_ NPs size are higher than γFe_2_O_3_ NPs). In fact, bactericidal effect is increased in nanoparticles with smaller size, due to cell uptake, intracellular distribution and interaction with biological macromolecules (in particular with microbial membranes) [45].

The antibacterial activity of both TiO_2_ and γFe_2_O_3_ is reported to mainly due to generation of free radicals and liberation of metal ion [46,47]. About γFe_2_O_3_, it has been found that ROS generation induces membrane depolarization, lipid peroxidation and DNA damage, while ion release negatively affects metabolic activities, cell homeostasis and protein functions [48,49]. Further, iron nanoparticles are able to penetrate into biofilms [50]. In terms of TiO_2_, its antibacterial effect is closely related to generation of ROS under UV-radiation or in dark condition in presence of oxidizing species [29,30,31]. It has also been reported that UV-independent antibacterial activity increases by the use of transition metal ions as dopants [47,51,52].

**Figure 7:**
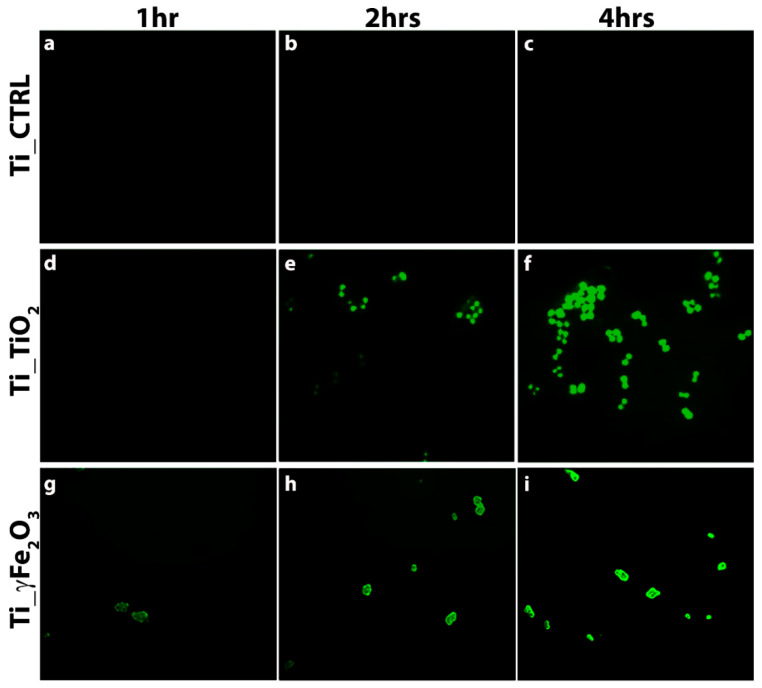
H_2_DCFD staining after 1, 2 and 4 h of incubation of *S. aureus* on Ti_CTRL (**a**–**c**), Ti_TiO_2_ (**d**–**f**) and Ti_γFe_2_O_3_ (**g**–**i**) scaffolds.

In order to verify the presence of intracellular ROS induced by the nano-functionalized scaffolds, 2′, 7’-dichlorodihydrofluorescein diacetate (H_2_DCFDA) staining was performed after 1, 2 and 4 h post bacteria incubation and fluorescence images detected. Specifically, ROS signal was evaluated in the early hours (1, 2 and 4 h), because ROS-positive cells decrease due to the reduction of viable cells [51]. Results are reported in Figure 7. It can be noticed that green fluorescence clearly appears in both Ti_TiO_2_, and Ti_γFe_2_O_3_ scaffolds after 2 h (Figure 7e,h) and increases after 4 h (Figure 7f,i). On the contrary, the Ti_CTRL does not show any ROS evidence (Figure 7a–c). This finding indicates the ROS presence in agreement with the literature data above mentioned.

The above results highlight that both Ti_TiO_2_ and Ti_γFe_2_O_3_ are able to match the double function to promote osteo-integration and inhibit bacterial invasion. To further investigate these aspects, we plan further studies focusing on the optimization of the nano-functionalization (in terms of NPs densities, surface charges) and to gain more insight on the regeneration and bactericidal mechanisms with in vitro studies to evaluate the specific genes and cellular pathways to be finally validated by in vivo study.

## 4. Conclusions

In this study, we prepared Ti scaffold nano-functionalized with TiO_2_ and γFe_2_O_3_ NPs by surface in situ growing method and their osteo-integrative/inductive capability and antimicrobial activity were assessed. Morphological characterization of both Ti_TiO_2_ and Ti_γFe_2_O_3_ scaffolds showed the presence of TiO_2_ NPs with dimensions between 300 nm and 800 nm in diameter spread onto Ti surface, while in the case of γFe_2_O_3_ both TEM and AFM measurements highlight the presence of NPs with a diameter in the range of 10–20 nm. Both Ti_TiO_2_ and Ti_γFe_2_O_3_ scaffolds exhibited good osteo-integration ability. In particular, a considerable increase of hADSCs cell growth after 7 days of culture equal to 2.3-fold for Ti_TiO_2_ and 2.6-fold for Ti_γFe_2_O_3_, compared to the control, was found. Further, an improved osteogenic differentiation was observed with an ALP activity increasing up to 17%. SEM analyses highlighted good cell adhesion and colonization over the scaffold porosities.

Good antibacterial activities were exhibited from both Ti_TiO_2_ and Ti_γFe_2_O_3_ scaffolds with a reduction of 99.4% and 99.9% of *S. aureus* colony number, respectively, compared to the not-treated sample. H_2_DCFDA staining investigation reveals the presence of ROS occurring in presence of both TiO_2_ and γFe_2_O_3_ nano-functionalization. These data prove that both Ti_TiO_2_ and Ti_γFe_2_O_3_ scaffolds are able to match the double function to promote osteo-integration and deplete bacterial invasion. Further in vitro investigations are planned to optimize the nano-functionalized coating (in terms of NPs densities, surface charges, etc.) and gain more insight on mechanisms inducing bone regeneration and antibacterial activity, before final validation by in vivo studies. Results here presented pave the way for future use of Ti_TiO_2_ and Ti_γFe_2_O_3_ biomimetic scaffolds to reconstruct large bone defect in orthopedic fields.

## Supplementary Materials

The following are available online at https://www.mdpi.com/article/10.3390/nano11102634/s1, Figure S1: Diffraction Analysis from TEM; Figure S2: DAPI cell count at D1 and D7; Figure S3: DAPI cell count (DAPI positive nuclei/field) at D1 and D7. Data are showed as mean ± standard deviation obtained on 20 fields.
