# Supplementary material for: Dual-Functional Nano-Functionalized Titanium Scaffolds to Inhibit Bacterial Growth and Enhance Osteointegration
Source: Nanomaterials (Basel). 2021 Oct 7;11(10):2634. doi: 10.3390/nano11102634 (PMC8537418; doi:10.3390/nano11102634)
Supplement: Supplementary file 1 [file nanomaterials-11-02634-s001.zip › nanomaterials-1394258-supplementary.pdf]

Supplementary Information

Dual-Functional Nano-Functionalized Titanium Scaffolds to Inhibit Bacterial Growth and Enhance Osteointegration

Giovanna Calabrese [1,*,†], Domenico Franco [1,†], Salvatore Petralia [2], Francesca Monforte [3], Guglielmo Guido Condorelli [3], Stefano Squarzoni [4,5], Francesco Traina [5,6] and Sabrina Conoci [1,7,*]

1 Department of Chemical, Biological, Pharmaceutical and Environmental Sciences, University of Messina, 98166 Messina, Italy; dfranco@unime.it
2 Department of Drug Science and Health, University of Catania, 95125 Catania, Italy; salvatore.petralia@unict.it
3 Department of Chemical Science, University of Catania, 95125 Catania, Italy; marzia.monforte@gmail.com (F.M.); guido.condorelli@unict.it (G.G.C.)
4 CNR—Institute of Molecular Genetics "Luigi Luca Cavalli-Sforza", 40136 Bologna, Italy; squarzoni@area.bo.cnr.it
5 IRCCS Istituto Ortopedico Rizzoli, 40136 Bologna, Italy; traina.francesco@gmail.com
6 Department of DIBINEM, University of Bologna, 40136 Bologna, Italy
7 Istituto per la Microelettronica e Microsistemi, Consiglio Nazionale delle Ricerche (CNR-IMM), Ottava Strada n.5, 95121 Catania, Italy
* Correspondence: gcalabrese@unime.it (G.C.); sconoci@unime.it (S.C.)
† These authors contributed equally to this work.

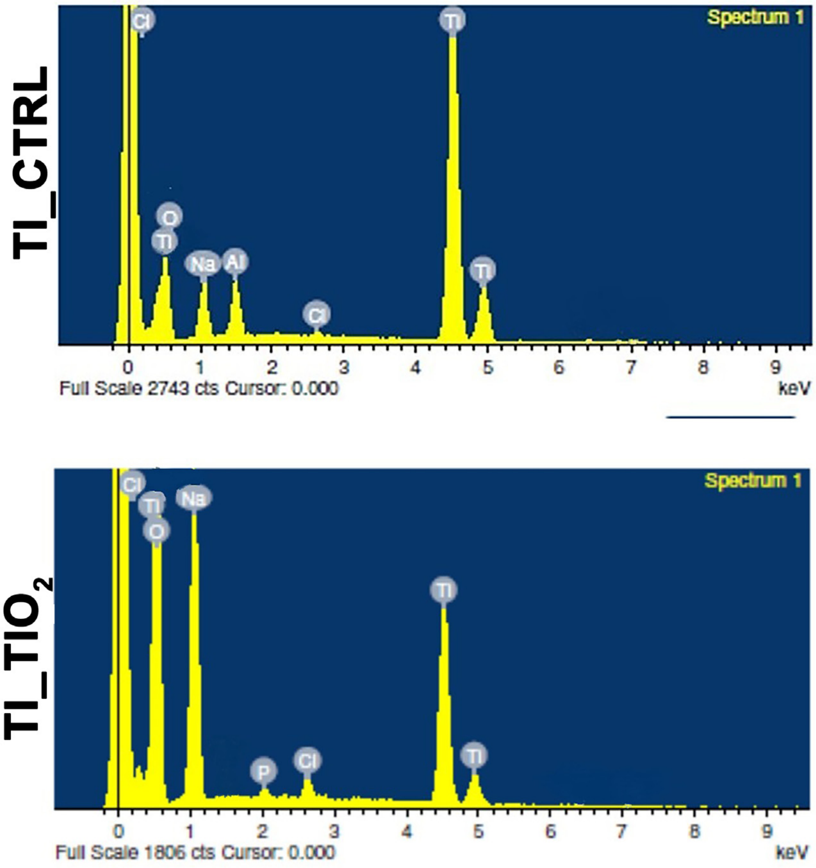

Figure S1. EDX analysis of Ti_CTRL vs Ti_TIO$_2$ scaffolds.

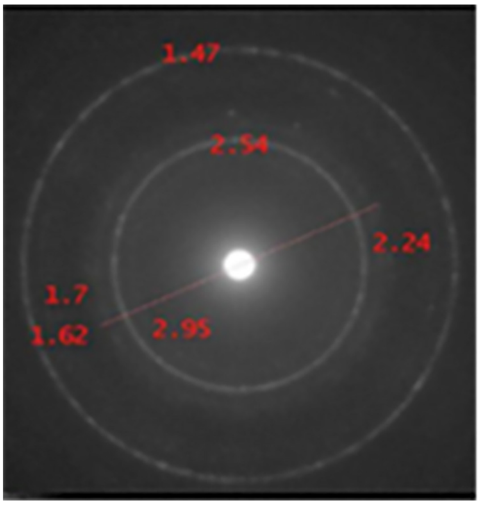

Figure S2. Diffraction Analysis from TEM

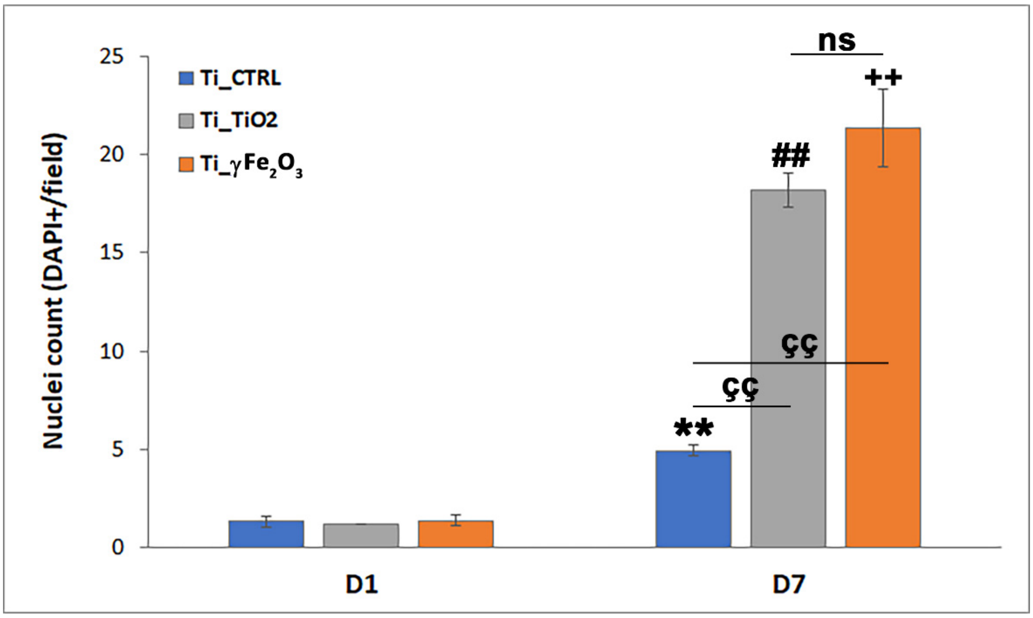

Figure S3. DAPI cell count (DAPI positive nuclei/field) at D1 and D7. Data are showed as mean ± standard deviation obtained on 20 fields. **,$^{\#\#}$,^{çç},$^{++}$p<0.01 show significant differences between the different time points and scaffolds, as reported by the Holm post-hoc test.
